# Comparison of the Pediatric Cochlear Implantation Using Round Window and Cochleostomy

**DOI:** 10.22038/ijorl.2019.37313.2219

**Published:** 2020-01

**Authors:** Masoud Naderpour, Zohreh Aminzadeh, Yalda Jabbari Moghaddam, Bita Pourshiri, Aida Ariafar, Afarin Akhondi

**Affiliations:** 1 *Department of Otorhinolaryngology, Tabriz University of Medical Sciences, Tabriz, Iran.*; 2 *Department of Pediatrics, Tabriz University of Medical Sciences, Tabriz, Iran.*

**Keywords:** Cochlear Implantation, Cochleostomy, Hearing Preservation, Round Window

## Abstract

**Introduction:**

Cochlear implantation (CI) is now regarded as a standard treatment for children with severe to profound sensor neural hearing loss. This study aimed to compare the efficacy of the round window approach (RWA) and standard cochleostomy approach (SCA) in the preservation of residual hearing after CI in pediatric patients.

**Materials and Methods:**

This double-blind randomized controlled trial was conducted on 97 pediatric patients receiving CI with 12-month follow-up. The study population was divided into two groups according to the surgical approaches they received, including RWA and SCA. Consequently, the patients were evaluated based on the Categories of Auditory Performance scale (CAP) and Speech Intelligibility Rating (SIR) test 45-60 days and 3, 6, 9, and 12 months post-surgery.

**Results:**

The CAP and SIR mean scores increased in both groups during the 12-month follow-up. This upward trend was significant in both groups (P<0.001). There was no significant difference between the two treatment groups in any of the follow-up stages regarding the CAP mean score. The mean SIR score (P=1.14±0.40) was significantly higher in the RWA group 3(P=0.001), 6(P=0.008), and 9(P=0.006) months after the surgery. However, there was no significant difference between the RWA and SCA groups, regarding 1-year SIR (P=0.258).

**Conclusion:**

The CI with either RWA or SCA could improve hearing and speech performance in pediatric patients. Although mid-term speech intelligibility was better for RWA, there was no significant difference in the 1-year outcome between these two methods.

## Introduction

Continuous developments of cochlear implantation (CI) over 50 years have revolutionized the rehabilitation of patients with hearing impairment (1). The CI has been increasingly used to treat serious to deep sensorineural hearing loss (SNHL) (2). Previously, CI was viewed as a destructive inner ear procedure (3). Recently, various methods have been described by different researchers for cochlear electrode implantation (4-9). Over years, these surgical techniques have evolved to limit the intracochlear trauma and enhance electrode placement within the Scala tympani, regarding spiral ganglion neurons (10). Two atraumatic methods, namely round window approach (RWA) and "soft surgery" cochleostomy, have been used more commonly in recent years. The cochleostomy method was first described in 1993 by Lenhardt (3). A standard cochleostomy approach (SCA) is performed by drilling anterior/inferiorly to the round window membrane (RWM) to achieve the Scala tympani (1). Although the RWA was the first global method for the electrode substitution of the CI, it was less commonly used due to inducing osseous spiral lamina trauma caused by its insertion angle or hard and straight electrodes (3). However, given the advancement of more changeable electrodes and the emergence of hearing preservation approaches, nowadays, RWA is considered a less traumatic method (2). 

Both surgical techniques could lead to intracochlear trauma caused by the way of electrode placement on the intracochlear structures (11). Another injury caused by these techniques are delayed lesions, defined as new fibrosis or osteosis which are secondary to the first trauma (12). In a new systematic revision evaluating more than 250 studies, there were no significant data to support either of the two abovementioned techniques (3).

Abased on the statistics, 1-3 neonates per 1,000 cases suffer from SNHL, while a greater proportion lose their hearing later during childhood. This condition interferes with the ordinary advancement of the auditory, speech, and language capabilities. As a result, it is vital to minimize the time and period of auditory shortage by reducing the interval between the bilateral deafness onset and hearing preservation interventions (13). Currently, CI is normally applied in the therapy of children with serious to profound SNHL (14).

While hearing preservation in adult population has been well-studied and several studies have addressed comparing SCA and RWA in this population, there are only a few reports in the pediatric literature (15). Therefore, this research was carried out to investigate the efficacy of RWI or SCA in the preservation of residual hearing after CI. 

## Material and Methods

*Study design and setting*

This randomized clinical trial was conducted at Tabriz Children Hospital, Tabriz, Iran. This study was confirmed by the Ethical Board of Tabriz University of Medical Sciences within 2015-2016. The study was conducted according to the Helsinki Declaration and informed written consent was gained from the guardians of all participants. The study was registered in a clinical trial registry under the number of IRCT20161215031429N3. 

***Participants***

A total of 104 patients with serious-to-deep SNHL who received cochlear implant between 2015 and 2016were involved in this study. Birth history and demographic information of the subjects were collected before the intervention. The inclusion criteria were SNHL diagnosis with an indication for CI and age of<18 years. On the other hand, the exclusion criteria were:1) CI re-operation, 2) cochlear malformation, 3) osteosclerosis, and 4) hearing loss due to autoimmune diseases.

***Randomization,*** ***patient enrolment, and blinding ***

The participants who enrolled in the study were randomly assigned into two groups of 52 members based on the surgical approaches they received, namely RWA and SCA, using sealed opaque envelopes. Moreover, the person in charge of data analysis and the physician who evaluated the participants and results were blind to group allocation.

***Surgical technique***

An experienced surgeon performed CI, and pre-surgery measures were standardized for all patients. The only treatment that was variable was the implant insertion technique. All Surgeries were carried out under general anesthesia with intraoperative facial nerve monitoring. The trans mastoid approach to the Scala tympani and round window was used in both methods. Cortical mastoidectomy was performed at the onset of the surgery, followed by a posterior tympanotomy (2). After the identification of the round window, the overhang of the niche was removed carefully to display the RWM. In the RWA group, a partial circumferential incision was made anteroinferiorly in the membrane (paracentesis). However, in the SCA group, cochleostomy was performed anteriorly to the round window niche. The full-length electrode insertion was performed over1min (16). Additionally, all participants received intravenous cephalosporin and 100 mg hydrocortisone (17).

Outcome measures 

To assess the CI outcome, the patients were evaluated using two scales, including categories of Auditory Performance scale (CAP) and Speech Intelligibility Rating (SIR) test. Moreover, all patients were asked to answer these tests in the follow-up visits performed 45-60 days and 3, 6, 9, and 12 months after the surgery. The CAP is an index consisting of eight categories arranged in order of increasing difficulty )^18^(.

The CAP could measure the speech intuition performance of children with implantation. It determines supraliminal performance, which shows daily hearing abilities in a more realistic way. This index includes a progressive scale of hearing intuitive ability differing from 0 “no recognition of the sounds available in the environment” to 7 “can use the telephone with a known listener”(19) (Table.1).

**Table 1:** Criteria for children’s performance on the categories of auditory perception scale (CAP)

Category	CAP
0	No awareness of environmental sounds
1	Awareness of environmental sounds
2	Response to speech sounds (e.g. ‘‘go’’)
3	Identification of environmental sounds
4	Discrimination of some speech sounds without lip-reading
5	Understanding of common phrases without lip-reading
6	Understanding of conversation without lip-reading
7	Use of telephone with known listener

The SIR was used to evaluate the speech intelligibility of the children after CI by assessing their everyday spontaneous speech. It consists of five performance categories ranging from “pre-recognizable words in spoken language” to “connected speech is intelligible to all listeners”(19) (Table.2).

**Table 2:** Speech Intelligibility Rating (SIR) criteria

Category	SIR
1	Pre-recognizable words in spoken language (the child’s primary mode of everyday communication may be manual)
2	Connected speech is unintelligible; intelligible speech is developing in single words when context and lip reading cues are available
3	Connected speech is intelligible to a listener who concentrates and lip-reads with in a known context
4	Connected speech is intelligible to a listener who has little experience of a deaf person’s speech; the listener does not need to concentrate unduly
5	Connected speech is intelligible to all listener’s; the child is understood easily in everyday contexts
	

***Statistical analysis***

Statistical analysis was calculated by SPSS software (version 22, SPSS Inc., IBM Corporation). The Kolmogorov-Smirnov test was used to evaluate the normality of the data distribution. Independent t-test and Chi-square test were used to compare the frequency distribution of the parametric and non-parametric data, respectively. Repeated measures design was used to compare the variables over time. P-values less than 0.05 were considered statistically significant.

## Results

For the purpose of achieving the eligibility, 118 patients were selected in the present study. A total of 14patients were excluded from the study due to the lack of eligibility (n=10), un willingness to participate in the study (n=3), and miscellaneous reasons (n=1). The remaining 104 patients were divided into two 52-member groups. In the follow-up sessions, 2 patients were left out from the SCA group due to the non-attendance. Finally, during the analysis, four patients were excluded from the SCA group, and one patient was removed from the RWA group because of missing data. As a result, 51 patients in the RWA group and 46 patients in the SCA group were analyzed (^Fig’s. 1^,2).

**Fig 1:**
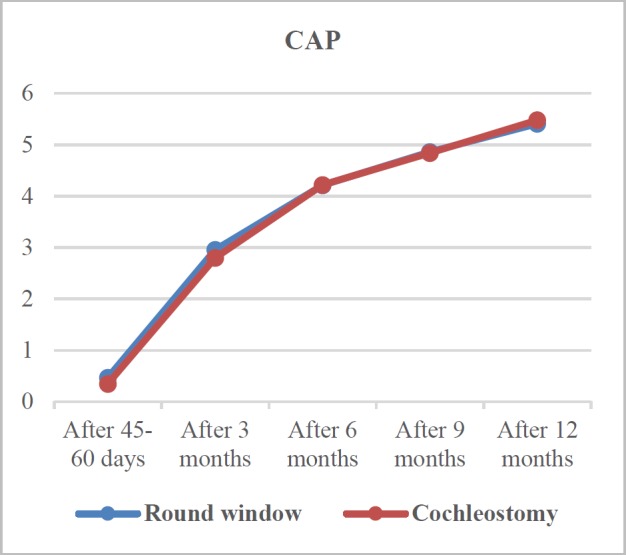
Comparison of CAP course in two treatment groups

**Fig 2:**
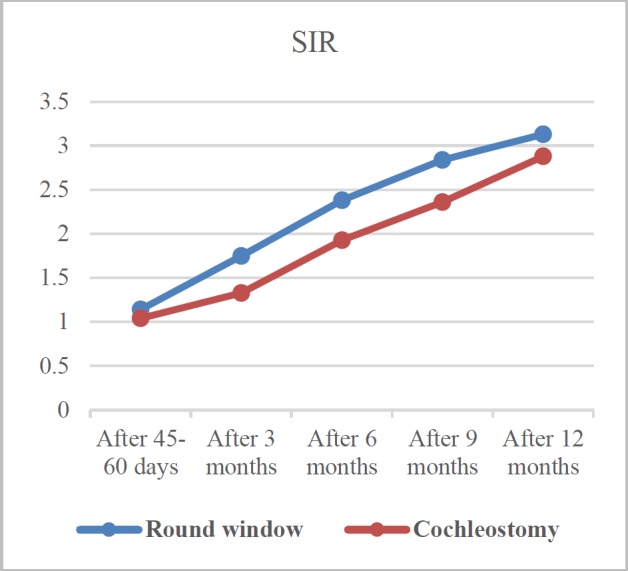
Comparison of SIR course in two treatment groups

***Patient Demographics***

At the baseline, no significant difference was observed between the two groups (Table.3). 

**Table 3:** Baseline Participants characteristics by Treatment Group

**Characteristic**	**RWA**	**SCA**	**P-value**
No.	51	46	
Age, No (%)			
<2 years	10 (20)	14 (30)	.139
2-5 years	31 (61)	29 (63)	
6-10 years	6 (12)	3 (7)	
>10 years	4 (8)	0 (0)	
Sex, No (%)			
Female	19 (37)	23 (50)	.225
Male	32 (63)	23 (50)	
Birth weight, No (%)^ *^			
<1.5 kg	1 (2)	2 (4)	.637
1.5-4 kg	48 (96)	42 (91)	
>4 kg	1 (2)	2 (4)	
Gestational age, No (%)^ *^			
Normal	42 (84)	41 (89)	.446
<37 weeks	7 (14)	4 (9)	
10 days before 42 weeks	0 (0)	1 (2)	
>42 weeks	1 (2)	0 (0)	
Implant type, No (%)			
Nucleus	48 (94)	45 (98)	.938
Advance	2 (4)	0 (0)	
MED-EL	1 (2)	1 (2)	
The cause of surgery, No (%)			
Infection	3 (6)	6 (13)	.301
Hyperbilirubinemia	29 (57)	27 (59)	>.999
Blood exchange transfusion	2 (4)	0 (0)	.497

*1 missing data

RWA: Round Window Approach

SCA: Standard Cochleostomy Approach

A total of 32 and 23 patients in the RWA and SCA groups were male, respectively. The patients aged 2-5 years constituted the largest group of our study (n=60; i.e., 31 cases in the RWA group and 29 cases in the SCA group). Regarding patient history, most of the subjects had a normal birth weight and gestational age (90 and 83 cases, respectively). The Nucleus CI 512 implant was used for CI the majority of our patients (42 cases in the RWA group and 45 cases in the SCA group). Regarding others, the Advance Hires 90K™ Advantage CI Hi Focus™ 1j Electrode implant was used for two patients in the RWA group, and the MED-EL SYNCHRONY implant was applied for one patient in each group. A total of 25 and 20 patients in the RWA and SCA groups with total hearing loss were referred for CI, respectively. The major cause of hearing impairment in our population was hyperbilirubinemia (56 cases; i.e., 29 and 27 patients in the RWA and SCA groups, respectively). The probable underlying causesin9 and 2 patients were infection and blood transfusion, respectively.

***Outcome measures***

Table 4 presents changes of the CAP and SIR mean scores during follow-up evaluations performed 45-60 days, and 3, 6, 9 and 12-months post-surgery. As noted, in both treatment groups, all outcomes improved throughout the course of the study. 

As Figure 1 depicts, the CAP mean score increased in both groups. In this regard, it enhanced from 0.47±0.58 to 5.41±0.95 and from 0.35 ± 0.74 to 5.4±1.21 within the time interval of 45-60 days to12 months post-surgery in the RWA and SCA groups, respectively. This enhancement in patient auditory performance based on CAP was significant for both approaches (both with P<0.001). There was no significant difference between the two treatment groups in none of the follow-up evaluations (all with P>0.05; Table.4). 

**Table 4:** Analysis of outcome measures by Treatment group

Outcome	Time of evaluation	RWA	SCA	P-value
CAP				
	After 45-60 days, Mean (Std. Deviation)	.47 (.58)	.35 (.74)	.079
	After 3 months, Mean (Std. Deviation)	2.96 (.92)	2.80 (1.05)	.108
	After 6 months, Mean (Std. Deviation)	4.21 (1.05)	4.22 (1.26)	.781
	After 9 months, Mean (Std. Deviation)	4.86(.89)	4.84(1.11)	.592
	After 12 months, Mean (Std. Deviation)	5.41 (.95)	5.48 (1.21)	.939
Within group P-value		<.001	<.001	
SIR				
	After 45-60 days, Mean (Std. Deviation)	1.14 (.40)	1.04 (.21)	.182
	After 3 months, Mean (Std. Deviation)	1.75 (.69)	1.33 (.63)	.001
	After 6 months, Mean (Std. Deviation)	2.38 (.84)	1.93 (.65)	.008
	After 9 months, Mean (Std. Deviation)	2.84(.83)	2.36(.74)	.006
	After 12 months, Mean (Std. Deviation)	3.13 (.83)	2.88 (.91)	.258
Within group P-value		<.001	<.001	

RWA: Round Window Approach       SCA: Standard Cochleostomy Approach CAP: Categories of Auditory Performance SIR: Speech Intelligibility Rating

As demonstrated in Figure 2, the SIR increased in both groups in the 12^th ^month of the surgery. The SIR mean scores were 1.14±.40 and 1.04±.21in the RWA and SCA groups, respectively, 45-60 days after the surgery. At the last evaluation stage (i.e., 12 months after CI), this score increased to 3.13±0.83 and 2.88±0.91 in the RWA and SCA groups, respectively. This upward trend was significant in both approaches (P<0.001; Table.4). Dissimilar to CAP, improvement in the SIR score was more prominent and greater in the patients receiving CI via the RWA and this difference was significant in the mid-term follow-up evaluations (P=0.001, P=0.008, and P=0.006 after 3,6, and 9 months, respectively). However, in the last evaluation (i.e., 1 year after surgery), no significant difference was observed between the two groups in this regard (Table.4).

## Discussion

There is an ongoing debate on opting for the most suitable method for CI between cochleostomy and RWAs. In the past, most surgeons preferred the cochleostomy approach (12). The main reason for this preference was the possibility of osseous spiral lamina trauma, along with larger surgical field and hard electrodes with RWA, leading to more residual hearing loss. Development of flexible and paramodular electrodes in recent years has arisen a renewed interest for RWA (17).

The aim of this research was to compare the hearing and speech performance of pediatric patients after CI via the RWA and SCA. In both approaches, patient performance was assessed using the CAP and SIR at different follow-up stages, including 45-60 days, and 3, 6, 9 and 12 months after the surgery. Patients in both groups showed an improvement at the end of the one-year follow-up.

In the patients receiving CI via the RWA, the CAP mean score improved significantly from short-term toward long-term evaluations increasing from 0.47 to 5.41. In the SCA group, CAP mean score was 15.66 times greater in the last evaluation, compared to that obtained in the first visit (5.48 vs. 0.35). The CAP mean score was slightly greater in the RWA group in both short and mid-term evaluations. However, no significant difference was observed between the two approaches in any of the study follow-up sessions.

Accordingly, CI implemented through the RWA or SCA improved patients CAP with no differences. 

The SIR mean score improved significantly from 1.75 to 3.13 and from 1.33 to 2.88 in the RWA and SCA groups, respectively. The SIR was greater in the RWA group, and this difference was significant in the mid-term assessments (3,6, and 9 months after the surgery); however, the final result of the two approaches did not differ significantly. Thus, although both surgical procedures could improve the SIR mean score, speech intelligibility improvement requires more time to compensate after implant insertion with cochleostomy.

Havenith et al. (3) reported that there is no valid evidence on the superiority of RWA or cochleostomy regarding hearing preservation after surgery. However, there were fewer number of patients with complete hearing loss after the RWA. According to a meta-analysis conducted by Santa Maria et al. (20) the cochleostomy approach was associated with better hearing preservation, compared to RWA. They suggested that this advantage might be due to the straight electrode insertion path into the cochlea in the cochleostomy approach. The reviewed studies mainly included (prospective) cohort studies and case series with heterogeneity regarding inclusion criteria and hearing stability identification; therefore, a probable risk of statistical bias could be expected.

In addition, in a meta-analysis performed by Santa Marinate al. (20), cochleostomy was described as any drilling to expose the Scala, and this definition consisted of extended RWA. Therefore, these two techniques were combined together for analysis, and finally, no conclusion could be reached on the technique (i.e., cochleostomy or extended RWA) with higher superiority. In recent years, several clinical trials and retrospective studies have been conducted to compare these two techniques. Cheng et al. (2), Hassepass et al. (17), and A dunk a et al. (21) reported clear advantages for both of the surgical approaches. Cheng et al. (2) demonstrated that in 40 adult patients who underwent CI, the RWA outcomes were comparable to those of cochleostomy, regarding electrode placement. They also observed no differences between the patients receiving CI via the RWA and cochleostomy when assessing speech perception based on tone, vowel, consonant, disyllable, and sentence perception 12 months after CI. In the mentioned study, similar to our research, 3-monthsentence perception was significantly greater in the RWA group (P=0.001); however, the 12-month outcome was comparable between the two groups. 

In a study carried out by Hassepass et al. (17), in 41 patients undergoing CI surgery, the assessment of speech perception in a quiet condition3-4months postoperatively showed significantly better results after both approaches, compared to the preoperative values. No significant difference was observed regarding the probability of complete low-frequency hearing loss between the cochleostomy and RWA group according to the audio logic data analysis.

A dunk a et al. (21) assessed hearing preservation and speech perception in 20 adult patients1-2 weeks, and 3, 6, and 12 months after the surgery. They concluded that CI implementation through RWA and cochleostomy rendered similar outcomes regarding both hearing preservation rates and speech perception 12 months after implantation. Briggs et al. investigated the presence of intracochlear trauma on 18fresh-frozen human temples (22). They reported no evidence of significant intracochlear trauma in neither RWA nor cochleostomy.

Some authors evaluated the surgical outcome with imaging techniques to compare RWA and SCA. Jiam et al. (23) evaluating 17 CI users with flat-panel computed tomography (FPCT), reported that the RWA resulted in shorter distances between the electrode and the modiolus. As a result, CI via the RWA could be a superior method as it might facilitate the placement of electrodes closer to the cochlear neural structures.

In another study performed by Jiam et al. (10), FPCT studies showed a higher probability of intercalary trauma with cochleostomy approaches. These results are in accordance with some reports in literature suggesting favorable outcomes with RWA. However, the sample size of these studies was small; therefore, further prospective studies are required to reach a clinical significance. On the contrary, Fan et al. (12) and Hassepass et al. (17) respectively using cone-beam computed tomography and FPCT reported no difference between the two CI approaches in terms of electrode position, insertion depth, and angle. 

Korsager et al. (16) studied the vestibular outcome after CI. They evaluated dizziness in patients with visual analogue scale and dizziness handicap inventory and found no statistically significant difference between the SCA and RWA regarding subjective dizziness after CI.

The present study involved the evaluation of hearing and speech performance in the pediatric patients receiving CI via the RWA or SCA with the use of CAP and SIR. The strengths of this study are its large sample size and a12-month follow-up period. However, the findings of this study should be explained with regard to its barriers. 

Primarily, patients hearing threshold levels were not evaluated for neither different frequencies nor depth of electrode insertion. Furthermore, due to the availability of one type of the implant (i.e., Nucleus CI512), it was not possible to examine the effects of other implant types. Additional studies with larger sample size and further variables (including insertion depth and angle and type of implant) are recommended in order to achieve more reliable results.

## Conclusion

As the findings of the present study indicated, the CI with both RWA and SCA can improve the hearing and speech performance in pediatric patients. However, mid-term speech intelligibility was better in the group subjected to RWA with no significant difference in the1-year outcome for these two methods.
